# Patients with *Trichinella spiralis* infection display unmodified antigen-specific immune response to SARS-CoV-2

**DOI:** 10.1590/0074-02760250044

**Published:** 2025-10-20

**Authors:** Ivana Mitic, Sofija Glamoclija, Natasa Radulovic, Ljiljana Sabljic, Sergej Tomic, Alisa Gruden-Movsesijan, Ljiljana Sofronic-Milosavljevic

**Affiliations:** 1 University of Belgrade, Institute for the Application of Nuclear Energy, Department for Immunology and Immunoparasitology, National Reference Laboratory for Trichinellosis, Belgrade, Serbia; 2 University of Belgrade, Institute for Biological Research “Sinisa Stankovic”, National Institute of the Republic of Serbia, Belgrade, Serbia; 3 University of Belgrade, Institute for the Application of Nuclear Energy, Department for Immunology and Immunoparasitology, Belgrade, Serbia

**Keywords:** Trichinella spiralis, SARS-CoV-2, immunomodulation, infection, T cells, B cells

## Abstract

**BACKGROUND:**

Through coevolution, helminths have developed immunomodulatory mechanisms that regulate exaggerated host immune responses and may influence immune responses to coinfections or vaccines. The coronavirus disease 19 (COVID-19) pandemic has raised concerns about how such infections might affect vaccine-triggered immune responses.

**OBJECTIVES:**

The aim of the study was to investigate how ongoing *Trichinella spiralis* infection affects the immune response to severe acute respiratory syndrome coronavirus 2 (SARS-CoV-2), in individuals already vaccinated or virus-primed, during Trichinella outbreak in Serbia.

**METHODS:**

Among 21 individuals who tested positive for anti-*Trichinella* antibodies, 15 were included in the study, which allowed for the first time to examine the impact of *Trichinella* infection on the humoral and cellular immune response to the SARS-CoV-2 using flow cytometry.

**FINDINGS:**

The results showed that *Trichinella* infection did not impair antibody production or cellular responses to SARS-CoV-2. Specifically, anti-SARS-CoV-2 antibodies and memory B cells remain unaffected, and T cells (CD4^+^ and CD8^+^) responded to SARS-CoV-2 antigens by generating pro-inflammatory cytokines.

**MAIN CONCLUSIONS:**

*Trichinella spiralis* infection does not disrupt the host’s humoral or cellular immune response to SARS-CoV-2, suggesting that the use of *Trichinella* antigens for the treatment of chronic inflammatory disorders, which is promising, will not affect the host’s ability to respond to future viral challenges.

With the global vaccination efforts during the coronavirus disease 19 (COVID-19) pandemic, questions have arisen about the potential impact of helminth infections on the immune response elicited by the use of new severe acute respiratory syndrome coronavirus 2 (SARS-CoV-2) vaccines, particularly in individuals residing in regions where the parasite infections are endemic. Vaccination against the SARS-CoV-2 virus, like natural infection, prompts early production of antibodies and establishes a durable memory B- and T-cell response.^1^ ^,^ ^2^ ^,^ ^3^ ^,^ ^4^ In patients with COVID-19, the seroconversion rates, which refer to the detection of antibodies against the SARS-CoV-2 virus following infection, were 90% or higher,^5^ ^,^ ^6^ and the production of pro-inflammatory cytokines such as interferon (IFN)-γ, tumour necrosis factor (TNF)-α, interleukin (IL)-2 and IL-6 was upregulated.^7^ Within the first month after infection, memory B- and T-cells specific to SARS-CoV-2 begin to emerge.^8^ The presence of SARS-CoV-2-specific memory B cells, as well as CD4^+^ and CD8^+^ T cells has been linked to milder symptoms and the establishment of protective immunity, largely owing to their ability to facilitate early viral clearance.^9^

A notable observation during the pandemic was the significantly lower incidence and mortality rates of COVID-19 in Africa and Latin America compared to more developed regions like North America, Western Europe, or South Asia.^10^ One possible explanation for this disparity could be the higher prevalence of parasite exposure within the populations of these countries. Interestingly, Adjobimey et al.^11^ have noted the inverse correlation between the presence of anti-*Ascaris* antibodies and the seriousness of COVID-19 symptoms, which implies that recent and ongoing *Ascaris* infections might lower the risk of severe COVID-19. Furthermore, in individuals co-infected with SARS-CoV-2 and filarial worm *Wuchereria bancrofti*, the presence of T cell hypoactivation may result in a comparatively milder progression of COVID-19, as reported by Mohamed et al.^12^ However, it is important to emphasise that helminths affect both immunopathological and protective bystander responses, thus the outcome of infection with helminths could be amplified susceptibility to other pathogens, with either attenuated or exacerbated pathology.^13^

Unlike viruses, helminths trigger both types of immune response, pro-inflammatory in the acute phase and anti-inflammatory during the chronic phase of infection. During long-lasting co-evolution with their hosts, helminths have developed mechanisms of immune modulation to restrain excessive immune responses and ensure their own survival, which means that they have to preserve the host’s organism as well. The beneficial effects of parasite infection and parasite-derived products on immune-mediated inflammatory disorders have been demonstrated through animal studies and promising clinical trials.^14^ ^,^ ^15^ Parasites’ excretory/secretory products are a complex mixture that includes proteins, glycans, lipids, nucleic acids, and extracellular vesicles. The application of these products has the potential to serve as a substitute for active helminth infections.^16^ ^-^ ^22^

*Trichinella spiralis* possess a unique characteristic among helminths, as it completes its entire life cycle within one host and creates a shelter for itself in the form of a completely new cell in the host’s organism, the nurse cell.^23^ This parasite inhabits different niches and acts both as an intestinal and intracellular parasite.^24^ In the form of muscle larvae, *Trichinella* reaches the intestine of the host (intestinal phase) through the consumption of raw, infected meat, mature into adults, which then occupy the enterocytes. The adults copulate and produce new-born larvae, which in migratory phase can invade various tissues, causing injury and inflammation, before entering muscle cells and transforming them into nurse cells. Nurse cell formation involves the fusion of an invaded muscle cell which undergoes de-differentiation, cell cycle re-entry, and arrest, with a satellite immature muscle cell that responds by proliferating and re-differentiating.^25^ As an encapsulated *Trichinella* species, *T. spiralis* muscle larvae reside in the nurse cell, surrounded by a collagen capsule, for a long period of time (muscle phase). *Trichinella* larvae balance pro- and anti-apoptotic mechanisms during this fusion.^25^ Initially, the infection triggers a Th1 immune response, but as the infection progresses, a Th2 response, responsible for parasite expulsion, becomes dominant. *T. spiralis* matures and reproduces before the Th2-mediated expulsion clears the adults from the gut. During the chronic muscle phase, the larvae remain protected from the host’s immune system within the nurse cell and communicate with the host through its excretory-secretory products (ES L1).^26^ ES L1 products consist of a diverse array of functional proteins, such as heat shock proteins, endonucleases, proteinases, protein kinases, proteinase inhibitors, superoxide dismutase, glycosidases, and extracellular vesicles, all of which play roles in various biological and immunological processes.^24^ ^,^ ^27^ ^,^ ^28^ The distinctive feature of the immune response, elicited in the chronic, muscle phase of the infection, is the heightened presence of anti-inflammatory and regulatory cytokines, IL-10 and TGF-β, and expansion of regulatory T cells (Treg), B cells and alternatively activated macrophages, which have the potential to suppress excessive Th1 and Th2 responses.^24^ ^,^ ^29^ ^,^ ^30^ ^,^ ^31^ ^,^ ^32^ Through induced immunomodulation, chronic helminth infection generally has the potential to dampen host’s immune responses, not confined solely to parasite antigens but to allergens and autoantigens as well.^13^ ^,^ ^33^ ^,^ ^34^ Additionally, it was shown that helminth infection could dampen immune response to coinfection and vaccines.^35^ ^,^ ^36^

A unique opportunity to investigate whether *T. spiralis* infection affects the immune response to unrelated antigens emerged during the COVID-19 pandemic. In March 2022, a trichinellosis outbreak occurred in Pozezeno village in Branicevo District, Serbia, an area endemic for trichinellosis,^37^ ^,^ ^38^ ^,^ ^39^ due to consumption of ham made from infected wild boar meat. Considering that the impact of *T. spiralis* infection on the immune response to viral infections or vaccines in humans is not well understood, we immediately initiated a small pilot study in aim to elucidate whether it influences the immune response to the SARS-CoV-2 virus currently circulating in the population. Out of the 27 individuals who consumed the ham and were suspected of having trichinellosis, 21 were tested positive for anti-*Trichinella* antibodies at the National Reference Laboratory for Trichinellosis (NRLT-INEP), Serbia. In collaboration with European Union Reference Laboratory for Parasites (EURLP), Istituto Superiore di Sanita (ISS) in Rome, Italy, NRLT-INEP successfully identified *T. spiralis* as a source of infection, with the worm burden of seven larvae per gram (LPG) of ham tissue consumed by the patients. To our knowledge this is the first study that examines the impact of ongoing infection with *T. spiralis* on humoral and cellular immune response to the SARS-CoV-2 virus.

## SUBJECTS AND METHODS

*Human subjects and study approval* - In early March 2022, individuals presented to the Primary Health Centre Veliko Gradiste, Serbia, with symptoms such as fever, periorbital oedema, and myalgia, consistent with the clinical features of trichinellosis.^40^ After clinical examination, along with haematological and biochemical analyses, suspected trichinellosis cases were referred to NRLT-INEP for examination of serum samples for anti-*Trichinella* antibodies. The diagnosis of trichinellosis was based on three primary criteria: clinical features (including at least three of the following six symptoms: fever, facial oedema, myalgia, diarrhoea, eosinophilia, and haemorrhages in the subconjunctival, subungual, or retinal regions); laboratory results (evidence of a *Trichinella*-specific antibody response); and epidemiological assessment (exposure to contaminated meat of a common source).^41^ Twenty-one patients met the case definition based on the diagnostic algorithm for acute *Trichinella* infection in humans.^40^ Fifteen individuals infected with *T. spiralis* who had previously experienced mild COVID-19 infection and/or vaccination against SARS-CoV-2 virus (referred to as the SARS-CoV-2 + TS group), as well as fifteen individuals without *T. spiralis* infection, making the control group, who had recovered from COVID-19 and/or had received vaccination (referred to as the SARS-CoV-2 group), provided peripheral blood samples after giving written informed consent. Blood samples from patients with trichinellosis were collected twice, six and 10 weeks post infection (p.i.), while blood samples from individuals of SARS-CoV-2 group were taken only once. Both SARS-CoV-2 + TS group and SARS-CoV-2 group were carefully matched in terms of age and their vaccination and/or recovery status with respect to COVID-19. All participants completed a questionnaire providing information on their vaccination status and history of COVID-19 infection. Furthermore, their immune status was verified and confirmed through a positive SARS-CoV-2-enzyme-linked immunosorbent assay (ELISA) test. Data on the course and treatment of acute trichinellosis were gathered from the patient’s medical histories.

*Serological analysis* - In March and April 2022, serum samples were collected from individuals infected with *T. spiralis* at the Primary Health Centre Veliko Gradiste, Serbia. Similarly, serum samples were collected from individuals in the control group at the Institute for the Application of Nuclear Energy-INEP, Belgrade, Serbia. The serum samples were subjected to testing for anti-*Trichinella* antibodies using the indirect immunofluorescence assay (IFA) (“FITC *Trichinella spiralis* Antibody Detection Kit”, INEP, Belgrade, Serbia). This test uses five-micron sections of paraffine-embedded *T. spiralis*-infected muscle tissue or isolated muscle larvae, which were deparaffinised in xylene, rehydrated through a graded alcohol series, rinsed and then incubated with serial dilutions of human serum samples. After 30 min of incubation, the sections were washed and treated with FITC-conjugated antibodies that target human IgG, IgA, and IgM. This fluorescent conjugate enables early detection of anti-*Trichinella* antibodies, including during the seroconversion phase. Non-specific binding was assessed using negative control serum. The slides were examined under ultraviolet microscopy (AXIO Imager A1, Carl Zeiss AG, Germany). A positive result was defined by the appearance of a distinct, bright apple-green fluorescence on the cuticle and within the stichosome of the larvae, while the absence of fluorescence indicated a negative result. Anti-*Trichinella* antibody titres equal to or higher than 1:40 were considered seropositive. For the detection of antibodies against SARS-CoV-2 in the examined serum samples, ELISA SARS-CoV-2 IgG (RBD - S protein) kit (INEP, Belgrade, Serbia) was used. The test’s specificity relies on immobilised recombinant SARS-CoV-2 proteins on an ELISA microtiter plate, which encompass the entire sequence of the receptor binding domain (RBD) within the S1 subunit of the spike protein. All samples were analysed in a single run to reduce potential intra assay variability. The results were evaluated semi-quantitatively and presented as an index, calculated based on optical density (OD) measurements using the formula: Index = (OD sample/OD positive control) × 90. Results with an index value below 15 were classified as negative while those with an index value of 20 or above were regarded as seropositive. Values of the index falling within the range of 15-20 were considered as the grey zone.

*PBMC culture and stimulation* - Peripheral blood mononuclear cells (PBMCs) were isolated from blood samples of patients with trichinellosis (SARS-CoV-2 + TS group) taken ~10 weeks after *T. spiralis* infection, as well as from individuals belonging to SARS-CoV-2 group, by density centrifugation Hystopaque-1077 (Sigma-Aldrich, Munich, Germany). PBMCs were resuspended in RPMI 1640 (Sigma-Aldrich, Munich, Germany) supplemented with 10% foetal calf serum (FCS) and antibiotics (penicillin at 100 units/mL, streptomycin at 0.1 mg/mL, and gentamicin at 0.08 mg/mL, all from Sigma-Aldrich, Munich, Germany). For T cell analysis, 1.5 × 10^6^ cells were seeded per well, while for B cell analysis 4 x 10^6^ of freshly isolated cells were used from the same donors. *T. spiralis* excretory-secretory products (ES L1) at a concentration of 10 μg/mL were used for T cell analysis following overnight incubation, prepared according to previously described method.^17^ Control cells were cultivated in medium without stimuli. Following this incubation, PBMCs were treated with a mixture of SARS-CoV-2 15-mer peptides covering the complete protein coding sequence of the surface or spike glycoprotein (“S”), nucleocapsid phosphoprotein (“N”) and membrane glycoprotein (“M”) (Miltenyi Biotech, Bergisch Gladbach, Germany), for T cell analysis, during 6h at a concentration of 1 μg/mL, all in the presence of Brefeldin A (1µM). As a positive control in these assays, the cells were stimulated with phytohemagglutinin (2 µg/mL), in the presence of Brefeldin A during 6 h. Following incubation at 37ºC with 5% CO_2_, cells were harvested and prepared for phenotype analysis using flow cytometry (FACS). Biotin-labelled RBD tetramers, in combination with Streptavidin in two different fluorochromes, were used to identify and phenotype RBD-specific B cells from both examined groups.

*Flow cytometry analysis* - Cells were incubated with primary antibodies in phosphate-buffered saline (PBS) containing 2% FCS and 0.1% NaN_3_ for 30 min at +4ºC. T cells were stained for surface markers with the following monoclonal antibodies: anti-CD4-AF700 (AF700-Alexa Fluor 700), anti-CD8-Pe-Cy7 (SK1) (Pe-*Phycoerythrin*, Cy7-Cyanine 7), anti-CD45RA-APC (APC-*Allophycocyanin*), anti-CCR7-PE (PE-*Phycoerythrin*), anti-CD3-PE-Dazzle (Dazzle 594) (all Biolegend, San Diego, CA, USA) antibodies. Cells were fixed using the flow cytometry fixation and permeabilisation kit I (R&D Systems, Minneapolis, MN, USA) for the purpose of intracellular staining with the following antibodies: anti-TNF-α-APC-Cy7 (*APC-Cy7-Allophycocyanin/Cyanine7*), anti-IL-10-APC, anti-IL-2-PerCP (PerCP-*Peridinin Chlorophyll Protein*), anti-IL-4-PerCP, anti-IL-13-PE and anti-IFNγ-FITC (L243) (FITC-Fluorescein Isothiocyanate) (Sony Biotechnology, San Jose, CA, USA). For immunophenotyping of B cells, PBMC were incubated with biotin-labelled RBD tetramers, for 1 h on + 4ºC. This treatment was followed by staining of cells using matched combinations of monoclonal antibodies (mAbs): anti-IgD-FITC, anti-CD27-PE, anti-CD3-PE-Dazzle, anti-CD19-PE-Cy7, as well as Streptavidin-APC and Streptavidin-APC-Cy7 (all Biolegend, San Diego, CA, USA). B cell subpopulations were classified to four main B cell subsets using the IgD/CD27 classification system (gated in CD19): naïve B cells (IgD^+^CD27^-^), pre-switch-memory (IgD^+^CD27^+^), post-switch memory (IgD^-^CD27^+^) and double-negative/exhausted (DN, IgD^-^CD27^-^ B cells. Single-labelled samples were used for compensation of signal overlap between the channels before each analysis. Non-specific fluorescence was determined according to isotype control antibodies and fluorescence minus one (FMO) control. For determination of non-specific background staining were used isotype-matched control monoclonal antibodies: immunoglobulin (Ig) G1 negative control-FITC (P3.6.2.8.1), IgG1 negative control-PE (P3.6.2.8.1), IgG1 negative control-PECy7 (P3.6.2.8.1), IgG1 negative control-APC (P3.6.2.8.1) (all eBioscience), IgG1 negative control-PerCP (MOPC-31C) (BD Biosciences), IgG2aκ negative control PE-Dazzle (MOPC-173), IgG1κ negative control-Alexa Fluor 700 (MOPC-21) (BioLegend, San Diego, CA, USA) and IgG1 negative control-APCCy7 (MOPC-21) (Abcam, Cambridge, UK). Doublets were excluded based on the forward scatter (FSC-H, FSC-A) and cell morphology by FSC-A and side scatter (SSC-A). Dead cells were analysed according to the fixable viability dye 620 (BD Biosciences, San Jose, CA, USA) in parallel samples and at least 100.000 or 500.000 cells from each sample for analysis of specific T and B cells respectively. Cell fluorescence was analysed with a BD Biosciences LSR II Flow Cytometer (Beckton Dickenson, San Jose, CA, USA). The cytometer is equipped with eight-colour channels (five from blue laser and three from red laser) and two physical parameters (FSC/SSC). Collected data was further assessed offline using software FlowJo VX (BD Biosciences, San Jose, CA, USA).

*Statistics* - All graphs were generated using GraphPad Prism 9.0.0 for Windows (GraphPad Software). All data was tested for outliers. The distribution of data (parametric vs. not parametric) was determined by the Shapiro-Wilk test. In case of normal distribution one-way analysis of variance (ANOVA) was used for comparison between groups, with Tukey post hoc test. Otherwise, a non-parametric Kruskal-Wallis with Dunn’s post hoc test was used. Significance was accepted when p < 0.05.

*Ethics* - All procedures were done in accordance with the ethical standards of the institutional and/or national research committee and with the 1964 Helsinki Declaration and its later amendments or comparable ethical standards. The study was approved by the Ethics Committee of the Institute for the Application of Nuclear Energy - INEP of the University of Belgrade (permission date: March 21st, 2022 in Belgrade, No. 02-468). Informed consent was obtained from all individual participants included in the study.

## RESULTS

**Fig. 1:**
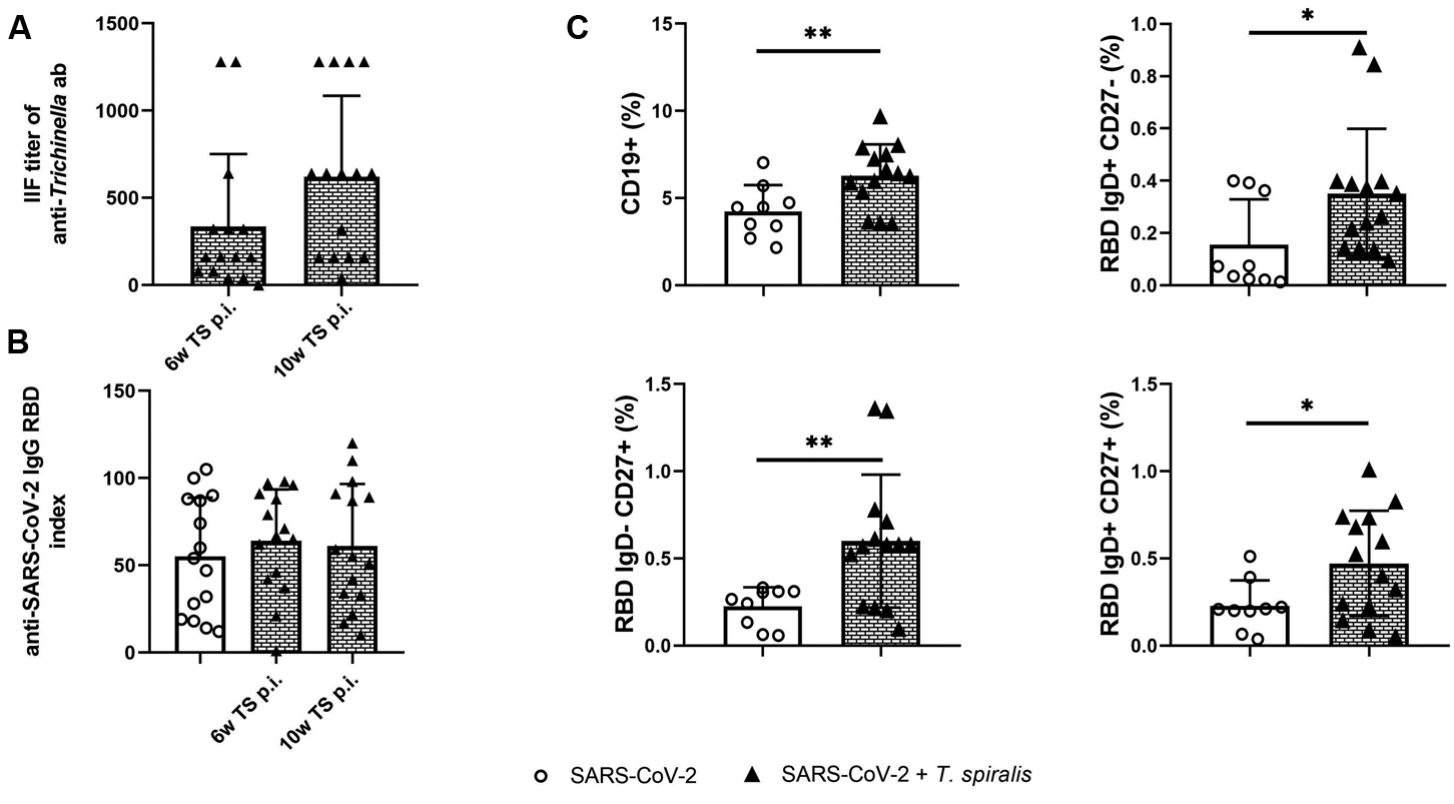
the indirect immunofluorescence assay (IFA) anti-*Trichinella spiralis* antibody titre (A) and the IgG severe acute respiratory syndrome coronavirus 2 (SARS-CoV-2) enzyme-linked immunosorbent assay (ELISA) index (B) evaluated in patients infected with *T. spiralis* (belonging to the SARS-CoV-2 + TS group), six and 10 weeks after *Trichinella* infection, and in patients belonging to the control group (SARS-CoV-2 group). Frequency of total CD19^+^ B cells and receptor-binding domain (RBD) spike protein (S) specific B cell subpopulations based on IgD/CD27 expression in SARS-CoV-2 + TS group [individuals infected with *T. spiralis* who had previously experienced mild coronavirus disease 19 (COVID-19) infection and/or vaccination against SARS-CoV-2 virus] and SARS-CoV-2 group [individuals without *T. spiralis* infection, who had recovered from COVID-19 and/or had received vaccination (control group)] (C). ^*^p < 0.05, ^**^p < 0.01.

*Serological findings* - A total of fifteen patients infected with *T. spiralis* (SARS-CoV-2 + TS group) were included in the study, with ages ranging from 21 to 63 years and an average age of 49.67 years (Table). All experienced a mild form of trichinellosis without any complications, and there were no hospitalised patients. In the control group which consisted only of individuals who had either recovered from COVID-19 or received the SARS-CoV-2 virus vaccine, or both, the age range was from 28 to 68 years with the average age of 46.40 years [Supplementary data (Table)]. In terms of gender distribution, 80% of individuals in both groups were male, 20% were female. Within the SARS-CoV-2 + TS group, fourteen patients tested positive for anti-*Trichinella* antibodies in the initial serum sample, using the IFA test. Four patients exhibited a weak positive result, with titres of either 1:40 or 1:80, six showed stronger antibody titres ranging from 1:160 to 1:320, while three patients displayed even higher titres of 1:640 and 1:1280 (Fig. 1A). In the second serum sample all patients tested positive for anti-*Trichinella* antibodies. Ten individuals showed the increased titres of anti-*Trichinella* antibodies, four persons maintained the same titre as in the initial sample, while patient, tested negative in the initial serum sample, was tested positive (Fig. 1A). As anticipated, none of the participants in the control (SARS-CoV-2) group yielded positive results for *Trichinella* during testing [Supplementary data (Table)].

**TABLE:** Epidemiological profile of trichinellosis patients with prior experience of coronavirus disease 19 (COVID-19) infection and/or vaccination against severe acute respiratory syndrome coronavirus 2 (SARS-CoV-2), along with serological findings

No	Gender	Age	Samples acquired	COVID 19 infection timeline	Vaccines against SARS-CoV-2 (time of last dose)	ELISA SARS-CoV-2 6/10 weeks p.i.^*^ (Positive > 20)	IFA *T. spiralis* 6/10 weeks p.i.^*^ Positive > 1:40)
1	M	60	22nd March/18th April 2022	Nov. 2020/March 2022	mRNA vaccine three doses (July 2021)	97/89	1:80/1:640
2	F	53		Nov. 2020/Feb. 2022	inactivated virus *vaccine* three doses (July 2021)	88/59	1:40/1:40
3	M	21		Jan. 2022	No	62/42	1:320/1:640
4	M	62		No	inactivated virus *vaccine* three doses (Nov. 2021)	67/51	1:160/1:320
5	M	51		Oct. 2021/Feb. 2022	inactivated virus *vaccine* two doses (March 2021)	71/110	1:1280/1:1280
6	M	30		Aug. 2021	No	1/10	1:640/1:1280
7	M	35		Sept. 2021	No	21/17	< 1:40/1:160
8	M	63		No	inactivated virus *vaccine* two doses + mRNA vaccine one dose (Jan. 2021)	65/55	1:160/1:160
9	F	58		No	inactivated virus *vaccine* two doses + mRNA vaccine one dose (Jan. 2021)	42/33	1:320/1:1280
10	F	60		No	mRNA vaccine three doses (Feb. 2021)	96/120	1:1280/1:1280
11	M	60		Jan. 2022	inactivated virus *vaccine* two doses + mRNA vaccine one dose (Feb. 2021)	79/98	1:40/1:160
12	M	47		Jan. 2021	No	37/34	1:80/1:160
13	M	61		No	inactivated virus *vaccine* three doses (Jan. 2021)	91/91	1:160/1:640
14	M	43		Jan. 2022	inactivated virus *vaccine* two doses (April 2021)	46/22	1:320/1:640
15	M	41		Jan. 2021	mRNA vaccine two doses (Oct. 2021)	98/87	1:160/1:640

*6/10 weeks p.i. - patient serum sample taken 6/10 weeks post infection with *Trichinella spiralis*; ELISA: enzyme-linked immunosorbent assay; IFA: indirect immunofluorescence assay.

Initial sera samples from SARS-CoV-2 + TS group and control, SARS-CoV-2 group, were tested for anti-SARS-CoV-2 antibodies in anti-RBD-S ELISA (Fig. 1B). Thirteen individuals from SARS-CoV-2 + TS group showed detectable levels of anti-RBD protein antibodies with a mean value of 64.07 ± 29.54, while the two patients tested negative (according to anamnestic data, they were not vaccinated, but recovered from a mild COVID-19 infection seven months earlier). Analysis done in sera samples taken 10 weeks p.i. revealed similar results (Fig. 1B). In the control, SARS-CoV-2 group, all participants were tested positive for anti-RBD antibodies with a mean value 55.2 ± 33.56.

*B cell immune response* - The overall percentage of CD19 positive B cells was significantly higher in individuals from SARS-CoV-2 + TS group compared to control SARS-CoV-2 group (Fig. 1C). The frequencies of B cell subsets were defined by the expression of IgD and CD27, universal marker for human memory B cells. The gating strategy for phenotyping RBD-specific B cell is shown in Supplementary data (Fig. 1). Significantly higher proportion of RBD S specific IgD^-^CD27^+^ B cells (class-switched memory B cells) and IgD^+^CD27^+^ B cells (non-switched memory/ B cells) was observed in SARS-CoV-2 + TS group compared to SARS-CoV-2 control group (Fig. 1C). The frequency of IgD^+^CD27^-^ naïve B cell population in the peripheral blood of subjects infected with *T. spiralis*, was also significantly elevated compared to controls (Fig. 1C).

**Fig. 2:**
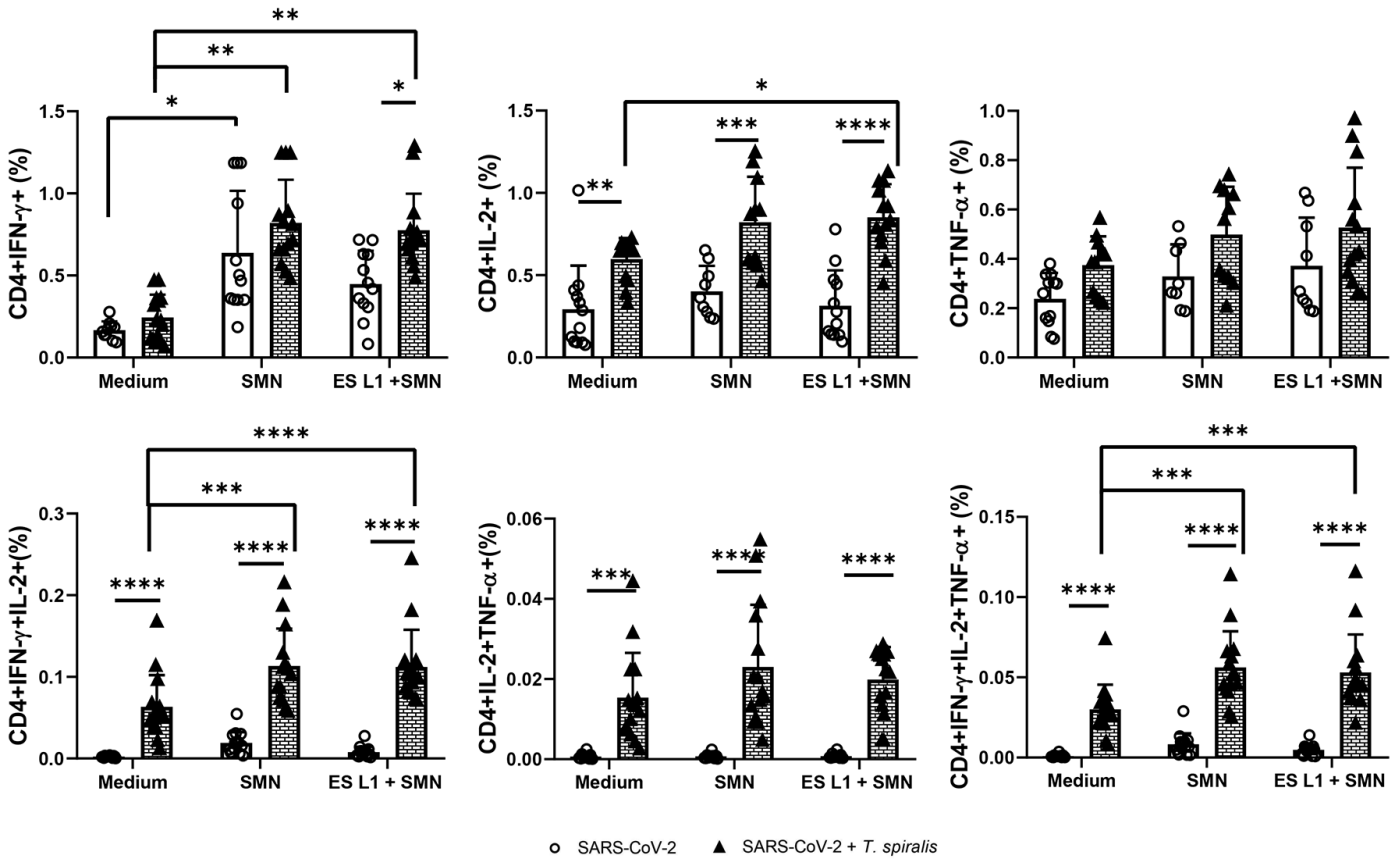
frequency of CD4^+^ cells expressing interferon (IFN)-γ, tumour necrosis factor (TNF)-α, or interleukin(IL)-2 and multifunctional CD4^+^ cells expressing two or three cytokines (IFN-γ, TNF-α, IL-2) as a proportion of total CD4^+^ T cells in severe acute respiratory syndrome coronavirus 2 (SARS-CoV-2) + TS group [individuals infected with *Trichinella spiralis* who had previously experienced mild coronavirus disease 19 (COVID-19) infection and/or vaccination against SARS-CoV-2 virus] and the SARS-CoV-2 group [individuals without *T. spiralis* infection, who had recovered from COVID-19 and/or had received vaccination (control group)] after incubation with SARS-CoV-2 viral proteins, with or without ES L1 *T. spiralis* (*T. spiralis* excretory-secretory products) stimulation. ^*^p < 0.05, ^**^p < 0.01, ^***^p < 0.001, ^****^p < 0.0001.

**Fig. 3:**
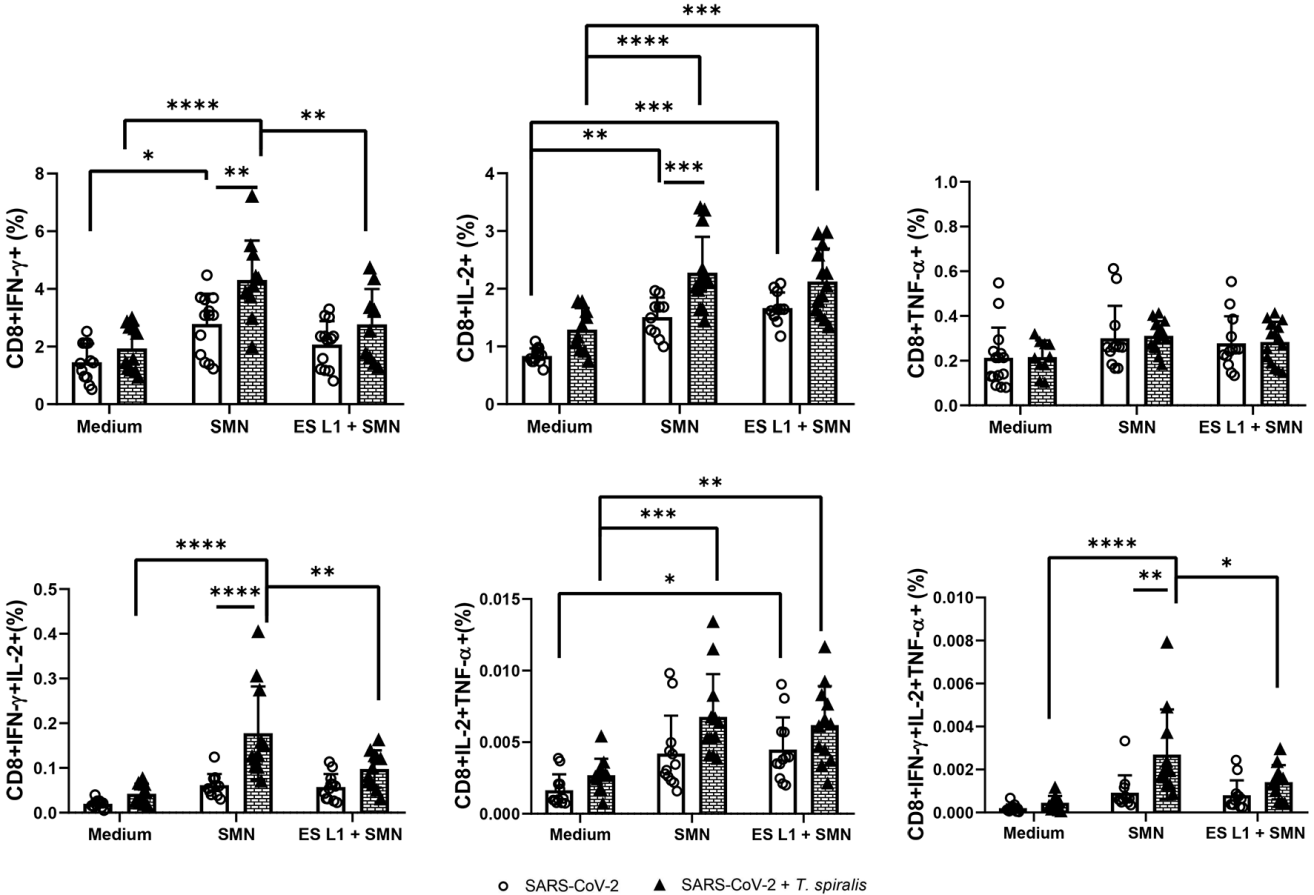
frequency of CD8^+^ cells expressing interferon (IFN)-γ, tumour necrosis factor (TNF)-α, or interleukin (IL)-2 multifunctional CD8^+^ cells expressing two or three cytokines (IFN-γ, TNF-α, IL-2) as a proportion of total CD8^+^ T cells in severe acute respiratory syndrome coronavirus 2 (SARS-CoV-2) + TS group [individuals infected with *Trichinella spiralis* who had previously experienced mild coronavirus disease 19 (COVID-19) infection and/or vaccination against SARS-CoV-2 virus] and the SARS-CoV-2 group [individuals without *T. spiralis* infection, who had recovered from COVID-19 and/or had received vaccination (control group)] after incubation with SMN [mixture of SARS-CoV-2 15-mer peptides covering the complete protein coding sequence of the surface or spike glycoprotein (“S”), nucleocapsid phosphoprotein (“N”) and membrane glycoprotein (“M”)] viral proteins and co-stimulation with SMN and ES L1 (*T. spiralis* excretory-secretory products) antigens of *T. spiralis*. ^*^p < 0.05, ^**^p < 0.01, ^***^p < 0.001, ^****^p < 0.0001.

*SARS-CoV-2-specific T cell response* - The overall percentage of CD3^+^ T cells was significantly higher (p < 0.001) in individuals from SARS-CoV-2 + TS group compared to control SARS-CoV-2 group. However, the CD4^+^/CD8^+^ ratio did not differ between groups [Supplementary data (Fig. 2)]. To assess the impact of *T. spiralis* infection on the ability of SARS-CoV-2-reactive memory CD4^+^ and CD8^+^ T cells to respond to re-stimulation with SARS-CoV-2 antigens, PBMCs were stimulated with SARS-CoV-2 peptides (mix of S, M and N antigens) alone, or with the combination of *T. spiralis* ES L1 and SMN antigens, followed by measurements of intracellular production of IFN-γ, IL-2 and TNF-α (Figs 2-3). Flow cytometric analysis of cell viability and the gating strategy for phenotyping cytokine-expressing T cells are presented in Supplementary data (Figs 2-3).

An increase in the percentage of SARS-CoV-2-specific CD4^+^ T cells expressing IFN-γ and IL-2 was observed in both groups of individuals, compared with the results obtained without stimulation, while no difference in the percentages of TNF-α expressing CD4^+^ T cells was observed. Comparison of SARS-CoV-2 + TS and SARS-CoV-2 groups revealed a trend of increase in the proportion of SARS-CoV-2-specific CD4^+^ T cells expressing IFN-γ, IL-2 or TNF-α in *Trichinella* infected group, albeit a statistically significant elevated percentage was only obtained in the case of CD4^+^ T cells expressing IL-2 (Fig. 2). Additionally, a robust and highly statistically significant increase in percentage of multifunctional SARS-CoV-2-specific CD4^+^ T cells was observed in SARS-CoV-2 + TS group. These cells showed co-expression of IL-2 and IFN-γ, IL-2 and TNF-α, and all three cytokines IFN-γ, IL-2, and TNF-α upon re-stimulation with SMN viral proteins, and this was not suppressed by ES L1 stimulation (Fig. 2).

Stimulation of PBMCs with SARS-CoV-2 peptides revealed the significantly elevated percentage of SARS-CoV-2-specific CD8^+^IFN-γ^+^ and CD8^+^IL-2^+^ T cells in SARS-CoV-2 + TS group compared to SARS-CoV-2 group. This increase in SARS-CoV-2 specific CD8^+^IFN-γ^+^ and CD8^+^IL-2^+^ T cells was also significant in both groups following SMN stimulation when compared to the medium-stimulated control groups (Fig. 3). The frequency of CD8^+^ T cells expressing TNF-α, upon SARS-CoV-2 peptide stimulation was slightly elevated in both groups compared to PBMC cultivated in medium alone, but there was no difference in the proportion of these cells between *T. spiralis* uninfected and infected group. Preincubation of PBMC with *T. spiralis* ES L1 products had no effect on the subsequent stimulation of these cells with SMN peptides, as reflected in unaltered percentage of CD8^+^ T cells expressing IL-2 and TNF-α, compared to SMN stimulation (Fig. 3). However, exposure to ES L1 significantly reduced the percentage of CD8^+^ T cells expressing IFN-γ in the SARS-CoV-2 + TS group. A statistically significant increase in multifunctional SARS-CoV-2-specific CD8^+^ T cells, co-expressing IL-2 and IFN-γ, IL-2 and TNF-α, as well as all three cytokines (IFN-γ, IL-2, and TNF-α), was observed in the SARS-CoV-2 + TS group following stimulation with SMN antigens compared to non-stimulated control cells. Additionally, exposure of T cells to ES L1 products before SMN stimulation reduced the percentage of CD8^+^ IFN-γ^+^ T cells, as well as multifunctional SARS-CoV-2-specific CD8^+^ T cells co-expressing IL-2 and IFN-γ, and IFN-γ, IL-2, and TNF-α in the SARS-CoV-2 + TS group, compared to these cells stimulated with SMN alone (Fig. 3). Overall, the data showed that CD4^+^ and CD8^+^ T cells in patients infected with *T. spiralis* respond to SMN antigens similarly to T cells from uninfected patients. Additional exposure of T cells to ES L1 products affected only the percentage of CD8^+^ IFN-γ^+^ T cells.

**Fig. 4:**
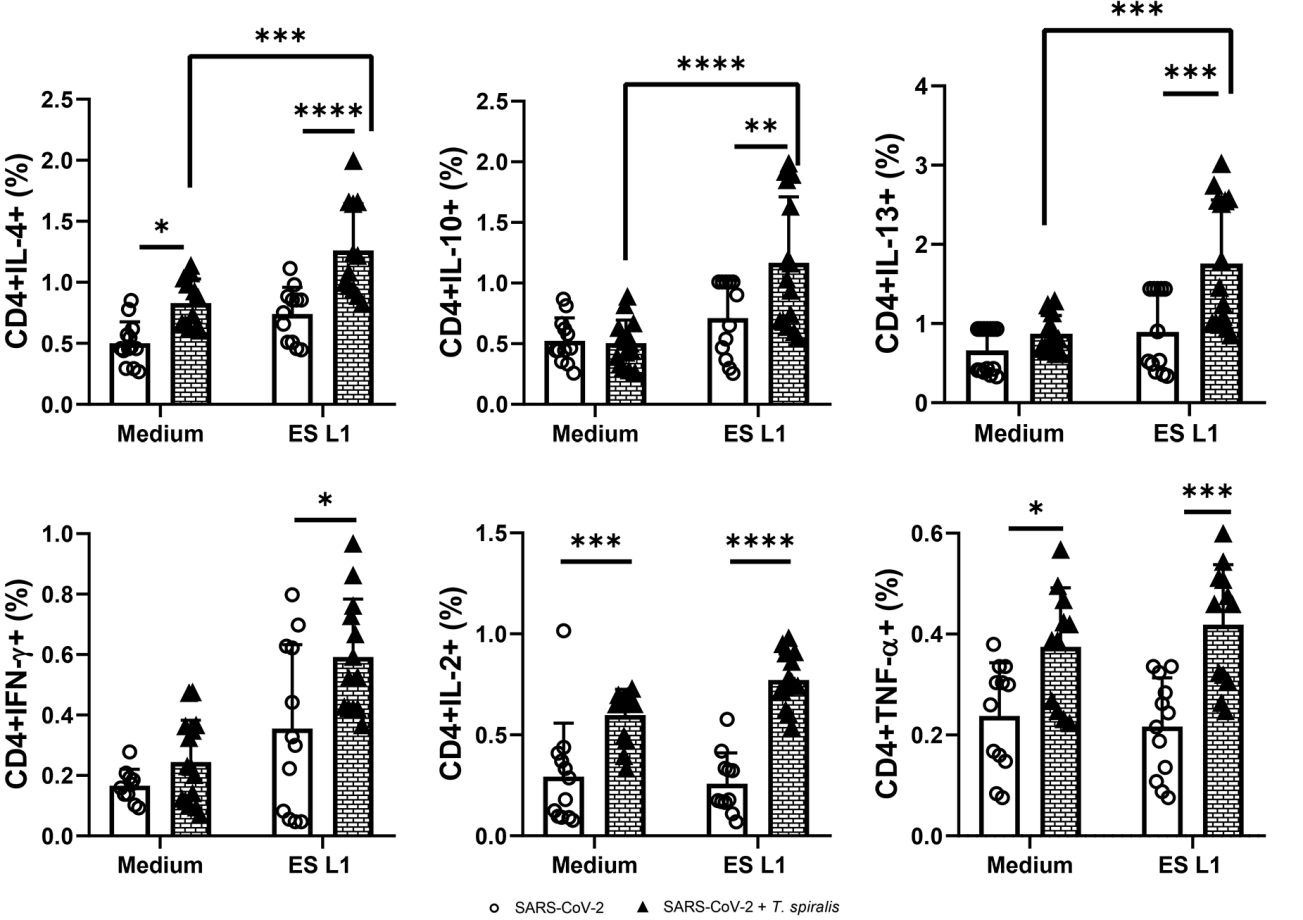
frequency of CD4^+^ cells expressing interleukin (IL)-4, IL-10, IL-13, interferon (IFN)-γ, tumour necrosis factor (TNF)-α and IL-2 as a proportion of total CD4^+^ T cells in severe acute respiratory syndrome coronavirus 2 (SARS-CoV-2) + TS group [individuals infected with *Trichinella spiralis* who had previously experienced mild coronavirus disease 19 (COVID-19) infection and/or vaccination against SARS-CoV-2 virus] and the SARS-CoV-2 group [individuals without *T. spiralis* infection, who had recovered from COVID-19 and/or had received vaccination (control group)] after incubation with ES L1 (*T. spiralis* excretory-secretory products) antigens of *T. spiralis.* ^*^p < 0.05, ^**^p < 0.01, ^***^p < 0.001, ^****^p < 0.0001.

**Fig. 5:**
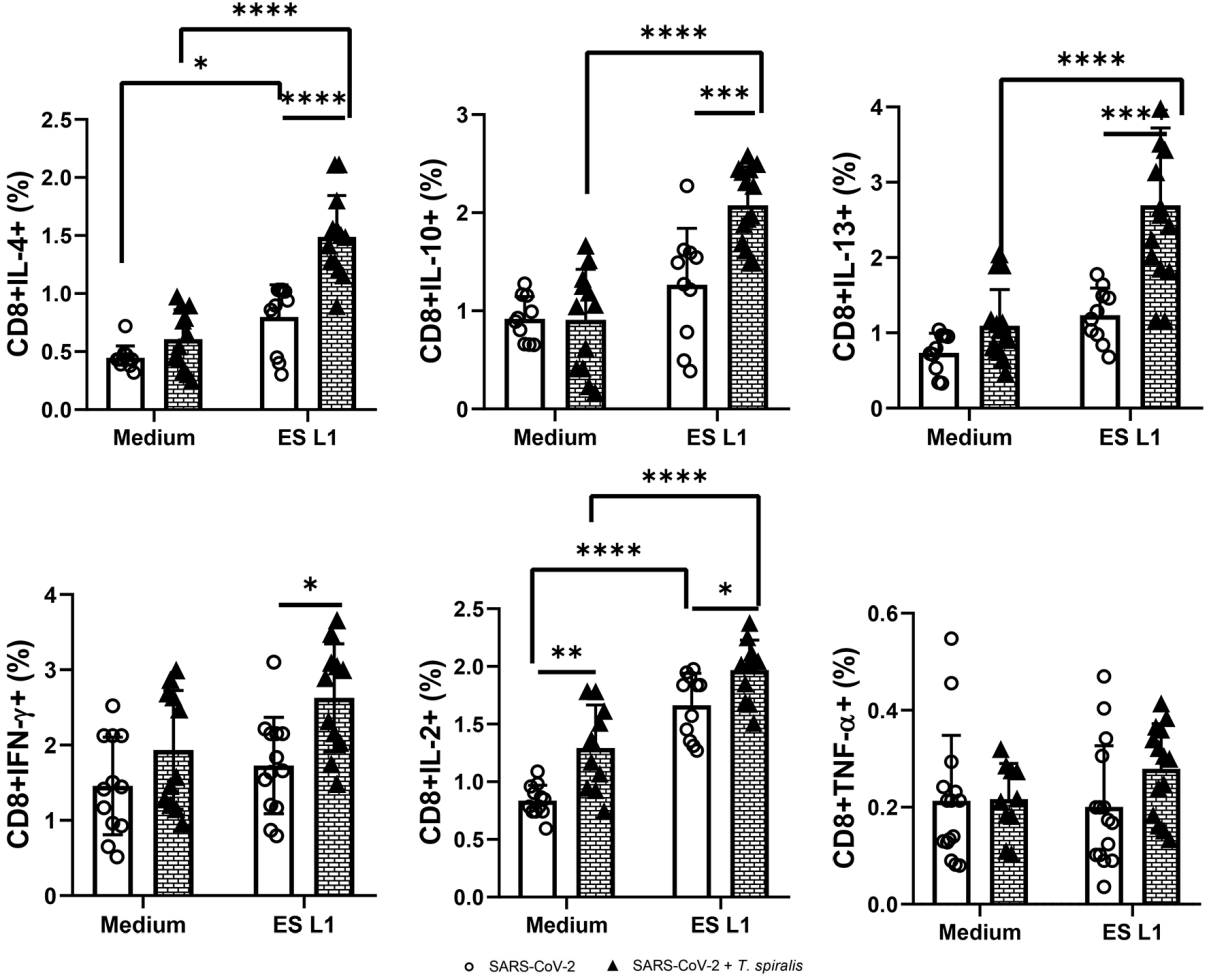
frequency of CD8^+^ cells expressing interleukin (IL)-4, IL-10, IL-13, interferon (IFN)-γ, tumour necrosis factor (TNF)-α and IL-2 as a proportion of total CD8^+^ T cells in severe acute respiratory syndrome coronavirus 2 (SARS-CoV-2) + TS group [individuals infected with *Trichinella spiralis* who had previously experienced mild coronavirus disease 19 (COVID-19) infection and/or vaccination against SARS-CoV-2 virus] and the SARS-CoV-2 group [individuals without *T. spiralis* infection, who had recovered from COVID-19 and/or had received vaccination (control group)] after incubation with ES L1 (*T. spiralis* excretory-secretory products) antigens of *T. spiralis*. ^*^p < 0.05, ^**^p < 0.01, ^***^p < 0.001, ^****^p < 0.0001.

*Trichinella spiralis-specific T cell response* - The response of *T. spiralis*-specific T cells in individuals from SARS-CoV-2 + TS group was evaluated by re-stimulation of PBMCs with *T. spiralis* ES L1 products, using the SARS-CoV-2 group as a negative control. The expression of anti-inflammatory cytokines, IL-4, IL-13 and IL-10, as well as pro-inflammatory cytokines IFN-γ, TNF-α and IL-2, within CD4^+^ and CD8^+^ T-cell subsets was analysed (Figs 4-5).

Among *T. spiralis*-infected patients, stimulation with ES L1 products led to a statistically significant rise in the frequency of CD4^+^ T cells expressing IL-4, IL-10, and IL-13 compared to the uninfected group. Additionally, there was also significant increase in the expression of pro-inflammatory cytokines, IFN-γ, TNF-α and IL-2 in SARS-CoV-2 + TS group, with or without stimulation with ES L1 (Fig. 4).

In patients infected with *T. spiralis,* there was a significant increase in the percentage of CD8^+^ cells, producing IL-4, IL-10, IL-13, as well as IL-2 upon ES L1 stimulation compared to cells cultivated in medium alone, without stimuli, and also compared to the proportion of these cells in SARS-CoV-2 group (Fig. 5). Judged by the obtained results, *Trichinella*-specific T cell response was not impaired in patients infected with *T. spiralis,* who had recovered from COVID-19 and/or received the SARS-CoV-2 vaccine. An intriguing observation was the existence of the response to ES L1 among cells from the uninfected SARS-CoV-2 group, which was reflected in an elevated percentage of CD8^+^ cells expressing IL-4 and IL-2, suggesting that ESL1 antigens do not just act as antigens, but as an immunomodulator.

## DISCUSSION

Numerous reports, especially from helminth-endemic regions, have recorded that parasites can suppress the production of antibodies in response to different pathogens and vaccines.^42^ ^-^ ^49^ Tweyongyere et al.^42^ conducted an observational study which revealed that *Schistosoma mansoni* infection correlated with a reduced measles-specific IgG response and lower likelihood of reaching protective IgG levels after immunisation in three- to five-year-old children, but the response was enhanced with praziquantel treatment. The lower response to measles vaccination among schoolchildren were also discovered by Nono et al.^43^ However, research suggests that parasite infections do not uniformly weaken vaccine-induced immune responses. Accordingly, a study investigating the impact of malaria and helminthic infections on the HPV vaccine found no detrimental effect on the immune response to the HPV-16/18 vaccine in the presence of these infections.^47^ Whether the presence of helminth infection will affect the effectiveness of vaccination depends, among other things, on the nature of the helminth-host relationship. In children with concurrent whipworm *Trichuris trichura* infection, the antibody response to a malaria vaccine candidate was reduced, whereas roundworm *Ascaris lumbricoides* infection did not impact the vaccination response.^48^

Our study revealed a high concentration of IgG antibodies specific for the SARS-CoV-2 spike protein RBD in 13 out of 15 patients with trichinellosis (SARS-CoV-2+TS group), suggesting that the presence of *T. spiralis* infection does not interfere with the production of antiviral antibodies. Although RBD-specific antibodies may contribute to protection against reinfection, their production relies on the development of an efficient B cell response, supported by an effective T-cell response.^50^ The presence of anti-SARS-CoV-2 antibodies six-12 months after infection and/or vaccination suggests the involvement of long-lived plasma cells, which have developed through the maturation of B cells during the immune response to the virus or vaccine. In addition to plasma cells, the primary response to antigens also generates memory B cells, which are capable of responding to repeated exposure to specific antigens and initiating a specific antibody response.

Throughout evolution, *Trichinella* has developed the ability to establish an immunoregulatory network that promotes its survival within the host while simultaneously protecting the host from excessive pro-inflammatory reactions. This immunomodulatory effect of *T. spiralis* has been studied in experimental animal models of allergies and autoimmune diseases, where it was shown that infection with this helminth could influence the beneficial outcome of the disease.^16^ ^,^ ^17^ ^,^ ^19^ ^,^ ^20^ ^,^ ^51^ ^-^ ^54^ The COVID-19 pandemic has underscored the significance of examining the immunomodulatory effects induced by helminth infections. Notably, evidence suggests that in regions where helminth infections are prevalent, individuals tend to experience milder clinical manifestations of COVID-19.^55^ Studies on animal models also revealed that helminth infection could exert beneficial effect on the outcome of viral infections.^56^ ^,^ ^57^ ^,^ ^58^ Infection with *T. spiralis* was found to attenuate the inflammatory lung damage caused by influenza virus but does not interfere with viral clearance mechanisms.^59^ On the other hand, helminth infection may impair antiviral immunity, as in case of *T. spiralis* and a murine norovirus co-infection.^60^

Given the already described influence of helminths, including *T. spiralis*, on immunity to viruses, we assumed that *T. spiralis* might also have an impact on immune response to SARS-CoV-2 infection or vaccination. Since mild COVID-19 infection and vaccination induce memory B and T cells, which are essential for protection against future severe SARS-CoV-2 infection,^61^ ^,^ ^62^ we investigated virus-specific memory B cells along with memory CD4^+^ and CD8^+^ T cells, which may have developed from previous exposure to viral antigens through COVID-19 infection and/or vaccination.

The increased frequency of total CD19^+^ B cells observed in *T. spiralis*-infected group can likely be attributed to the active *T. spiralis* infection. However, the significant increase in the percentage of RBD-specific IgD^-^CD27^+^ and IgD^+^CD27^+^ B cells, representing class-switched memory B cells and non-switched memory B cells respectively, observed in the SARS-CoV-2 + TS group compared to the SARS-CoV-2 group, was unexpected. Similarly, when assessing the impact of *T. spiralis* infection on the functional capability of SARS-CoV-2-specific memory T cells, we found that individuals infected with *T. spiralis* had a higher proportion of SARS-CoV-2-specific CD4^+^ and CD8^+^ T cells expressing IFN-γ, TNF-α, and IL-2, compared to the uninfected, control group. Given that the muscle phase of *T. spiralis* infection is typically associated with anti-inflammatory and regulatory responses, we initially anticipated a reduction in the inflammatory response in SARS-CoV-2 + TS group. Surprisingly, we found that this response was enhanced in *T. spiralis*-infected individuals. A possible explanation for this observed phenomenon may be the presence of epitopes on SARS-CoV-2 antigens and *T. spiralis* ES L1 components that share similar chemical and/or structural properties, potentially leading to lymphocytes cross-reactivity in epitope recognition. Consistent with this assumption is the finding that CD8^+^ T cells from the SARS-CoV-2 group responded to ES L1 products, even though these individuals were not infected with *T. spiralis*. Pathogens can share epitopes with unrelated pathogenic proteins, which may lead to a scenario where infection with one pathogen induces and/or alters an immune response against another unrelated pathogen.^63^ Number of studies revealed the presence of SARS-CoV-2-cross-reactive T cells, induced by previous encounters with coronaviruses or potentially other pathogens, and showed that these cells could influence the effectiveness of SARS-CoV-2-specific CD8^+^ and CD4^+^ T cell responses during infection and vaccination.^64^ ^,^ ^65^ ^,^ ^66^ Another investigation has indicated that *T. spiralis* antigens share similar epitopes with RSV. Not only did *T. spiralis*-specific antibodies recognise RSV antigens, but RSV-specific antibodies also cross-reacted with *T. spiralis* excretory-secretory antigens.^67^ ^,^ ^68^ In addition, in mice co-infected with both *T. spiralis* and RSV, *T. spiralis* infection resulted in a reduction in pro-inflammatory cytokines and a decrease in the presence of inflammatory cells in the lungs.^67^

During *T. spiralis* infection, components of ES L1 products affect host immune cells, acting either as antigens - triggering a specific immune response, or as immunomodulators - influencing responses to unrelated antigens, SARS-CoV-2 antigens in this study. Hence, using the ES L1 products as a pretreatment for SARS-CoV-2 SMN peptides stimulation of PBMC, was an attempt to test immunomodulatory effects of *T. spiralis* products on the responsiveness of SARS-CoV-2-specific T cells. Treatment with ES L1 products prior SMN stimulation did not alter the proportion of CD4^+^ and CD8^+^ T cells expressing IFN-γ, TNF-α, and IL-2, except for CD8^+^ T cells expressing IFN-γ. This indicates that the presence of ES L1 does not modulate TNF-α and IL-2 expression in these cells, while the impact on IFN-γ expression could be related to the elevated percentage of CD8^+^IL-10^+^ T cells upon exposure to ES L1. Taken together, these results clearly demonstrate that *T. spiralis* infection and ES L1 products of this parasite did not impair the functional capacity of SARS-CoV-2-specific memory CD4^+^ and CD8^+^ T cells originated from previous infection and/or vaccination.

Research studies have already demonstrated that a robust T cell response specific to SARS-CoV-2 is linked to less severe disease.^9^ ^,^ ^69^ ^,^ ^70^ This association suggests that both CD4^+^ and CD8^+^ T cells could have a significant role in managing and ultimately resolving an initial SARS-CoV-2 infection. The significant role played by multifunctional T cells in the immune response against the virus was highlighted by finding a greater proportion of SARS-CoV-2-specific CD4^+^ and CD8^+^ T cells, which produce two or three cytokines out of IFN-γ, TNF-ɑ, and IL-2 in patients with mild COVID-19.^71^ In our study, we observed a significantly higher percentage of multifunctional SARS-CoV-2-specific CD4^+^ T cells expressing two or three different proinflammatory cytokines (IL-2, IFN-γ, TNF-α) in patients infected with *T. spiralis*. This finding supports the assumption that *Trichinella* infection might not compromise the immune response to the virus but could potentially extend it, reducing the likelihood of developing a severe form of COVID-19.

Additionally, this study provided the opportunity to examine *T. spiralis*-specific T cell response during ongoing infection and evaluate whether it was in any way affected by previous COVID-19 illness or vaccination. Consistent with findings from other authors^72^ and our previously published data,^73^ ^,^ ^74^ this study revealed an increased proportion of CD4^+^ and CD8^+^ T cells expressing cytokines IL-4, IL-10 and IL-13 following stimulation of PBMC from *T. spiralis*-infected individuals with ES L1 products. However, while Gomez-Morales et al.^75^ showed a trend toward a decrease in CD4^+^ cells and increase in CD8^+^ cells during chronic muscle phase of *T. spiralis* infection, we did not observe such a trend, probably due to the fact that our research was conducted during the early muscle phase. Interleukin-10, IL-4, and IL-13 play a crucial role in promoting host tolerance to helminth infections. This is because IL-10 possesses anti-inflammatory properties, whereas both IL-4 and IL-13 are engaged in the process of tissue repair, which is essential to address the damage caused by parasitic worms including *T. spiralis*.^76^ The research conducted by Rolot et al.^57^ indicated that in the context of helminth infections, IL-4 can condition CD8^+^ T cells in a non-specific manner, resulting in a subsequent increase in the activation of antigen-specific CD8^+^ T cells, which, in turn, enhance the control of viral infections. This could be the additional explanation for the significantly elevated percentage of SARS-CoV-2-specific CD8^+^IFN-γ^+^ and CD8^+^IL-2^+^ T cells in *T. spiralis* infected individuals.

Our study showed that infection with *T. spiralis* did not suppress inflammatory response to challenge with SARS-CoV-2 antigens, but we cannot predict what would happen if coinfection occurred. What was observed is that simultaneous presence of both ES L1 and SMN antigens in PBMC culture, potentially simulating coinfection of the host with both *T. spiralis* and SARS-CoV-2, did not change the expression of pro-inflammatory cytokines within T cell compartment, compared to stimulation with viral proteins alone. Considering the results of other authors findings that robust T cell response specific to SARS-CoV-2 is linked to less severe disease,^9^ ^,^ ^69^ ^,^ ^70^ and our results showing significantly increased SARS-CoV-2-specific T cell response in *T. spiralis*-infected individuals, we can speculate that the immune response to a repeated encounter with SARS-oV-2 antigens would not be compromised and that coinfection could lead to the development of milder disease.

From our findings, we can conclude that *Trichinella* infection in humans does not inhibit either the production of RBD-specific antibodies or the effective cellular response to SARS-CoV-2 virus antigens. The level of anti-SARS-CoV-2 antibodies was not diminished and the same applies to SARS-CoV-2-specific B cells. T cells from an environment with an ongoing *Trichinella* infection showed the capacity to respond to repeated exposure to SARS-CoV-2 proteins (S, M, N) by producing pro-inflammatory cytokines IFN-γ, TNF-α and IL-2. To our current knowledge, this is the first human study that has assessed the impact of trichinellosis on the immune response to SARS-CoV-2 viral antigens. However, our study comes with several noteworthy limitations. Firstly, it was conducted during a single trichinellosis outbreak and involved a limited number of patients, which somewhat constrains the broader applicability of our findings. An additional limitation of the study is the variation in the patients’ vaccination regimens, including differences in vaccine types, combinations, sequencing, and the number of doses received. However, we had no control over these factors, as the experimental group self-selected by becoming infected with *Trichinella* during the outbreak. Secondly, due to the timing of PBMC sample collection, which occurred at approximately six to 10 weeks post-*T. spiralis* infection and at varying time points after COVID-19 infection and/or SARS-CoV-2 vaccination, we were unable to analyse the kinetics of immune responses across groups. Nevertheless, our study revealed that the majority of participants, with or without *T. spiralis* infection, did not exhibit a decrease in virus-specific CD4^+^ and CD8^+^ T cells, as well as memory B cells. Additionally, an inherent limitation lies in the *in vitro* stimulation of PBMCs with a viral peptide pool derived from the S, M, and N proteins, as this cannot be directly compared to whole virus stimulation of PBMCs. However, existing literature indicates that the S, M, and N proteins are codominant and recognised by 100% of COVID-19 cases.^77^ Given that our study participants were likely at the beginning of the chronic phase of trichinellosis, and that our previous research has shown that humoral immunity can last for more than a decade, probably as long as the larvae in the muscles remain viable,^78^ the next phase of our research could involve repeating this study several years after the trichinellosis episode to evaluate the recall response to SARS-CoV-2 antigens and possibly monitor the response to other viral vaccines, such as the seasonal influenza vaccine. This follow-up investigation would help confirm whether *T. spiralis* continues to maintain the balance and preserve the capacity of the host’s immune response for future potential encounters with the virus. Furthermore, the findings presented herein are encouraging, as they indicate that the administration of *Trichinella* antigens, suggested by our studies as potential therapeutics for chronic inflammatory disorders, may not impair the host’s capacity to elicit effective immune responses to future viral challenges, should these antigens be advanced to clinical evaluation.
