# Supplementary material for: Patients with Trichinella spiralis infection display unmodified antigen-specific immune response to SARS-CoV-2
Source: Mem Inst Oswaldo Cruz. 2025 Oct 20;120:e250044. doi: 10.1590/0074-02760250044 (PMC12543365; doi:10.1590/0074-02760250044)
Supplement: Supplementary file 1 [file 1678-8060-mioc-120-e250044-s.pdf]

TABLE

Epidemiological profile and serological findings of individuals in the control severe acute respiratory syndrome coronavirus 2 (SARS-CoV-2) group, who had prior experience of coronavirus disease 19 (COVID-19) infection and/or vaccination against SARS-CoV-2 virus

No	Gender	Age	Samples acquired	COVID 19 infection timeline	Vaccines against SARS-CoV-2 (time of last dose)	ELISA SARS-CoV-2 (Positive > 20)	IFA *T. spiralis* (Positive > 1:40)
1	M	68	April 2022	Dec. 2020/Aug. 2021	mRNA vaccine three doses (Aug. 2021)	55	< 1:40
2	F	47		Aug. 2020/Dec. 2021	inactivated virus vaccine three doses (Sep. 2021)	95	< 1:40
3	M	28		Feb. 2022	No	96	< 1:40
4	M	56		No	inactivated virus vaccine three doses (Sep. 2021)	53	< 1:40
5	M	46		April 2021/Jan. 2022	inactivated virus vaccine two doses (Aug. 2021)	26	< 1:40
6	M	28		Jan. 2021	No	31	< 1:40
7	M	29		Dec. 2021	No	100	< 1:40
8	M	57		No	inactivated virus vaccine two doses + mRNA vaccine one dose (Sep. 2021)	105	< 1:40
9	F	52		No	inactivated virus vaccine two doses + mRNA vaccine one dose (Oct. 2021)	25	< 1:40
10	F	54		No	inactivated virus vaccine three doses (Oct. 2021)	85	< 1:40
11	M	55		Feb. 2022	inactivated virus vaccine two dose + mRNA vaccine one dose (Oct. 2021)	71	< 1:40
12	M	42		Jan. 2022	No	20	< 1:40
13	M	56		No	inactivated virus vaccine three doses (Aug. 2021)	23	< 1:40
14	M	37		Feb. 2022	inactivated virus vaccine two doses (Sep. 2021)	21	< 1:40
15	M	38		Feb. 2021	mRNA vaccine two doses (Aug. 2021)	22	< 1:40

ELISA: enzyme-linked immunosorbent assay; IFA: indirect immunofluorescence assay.

CC BY

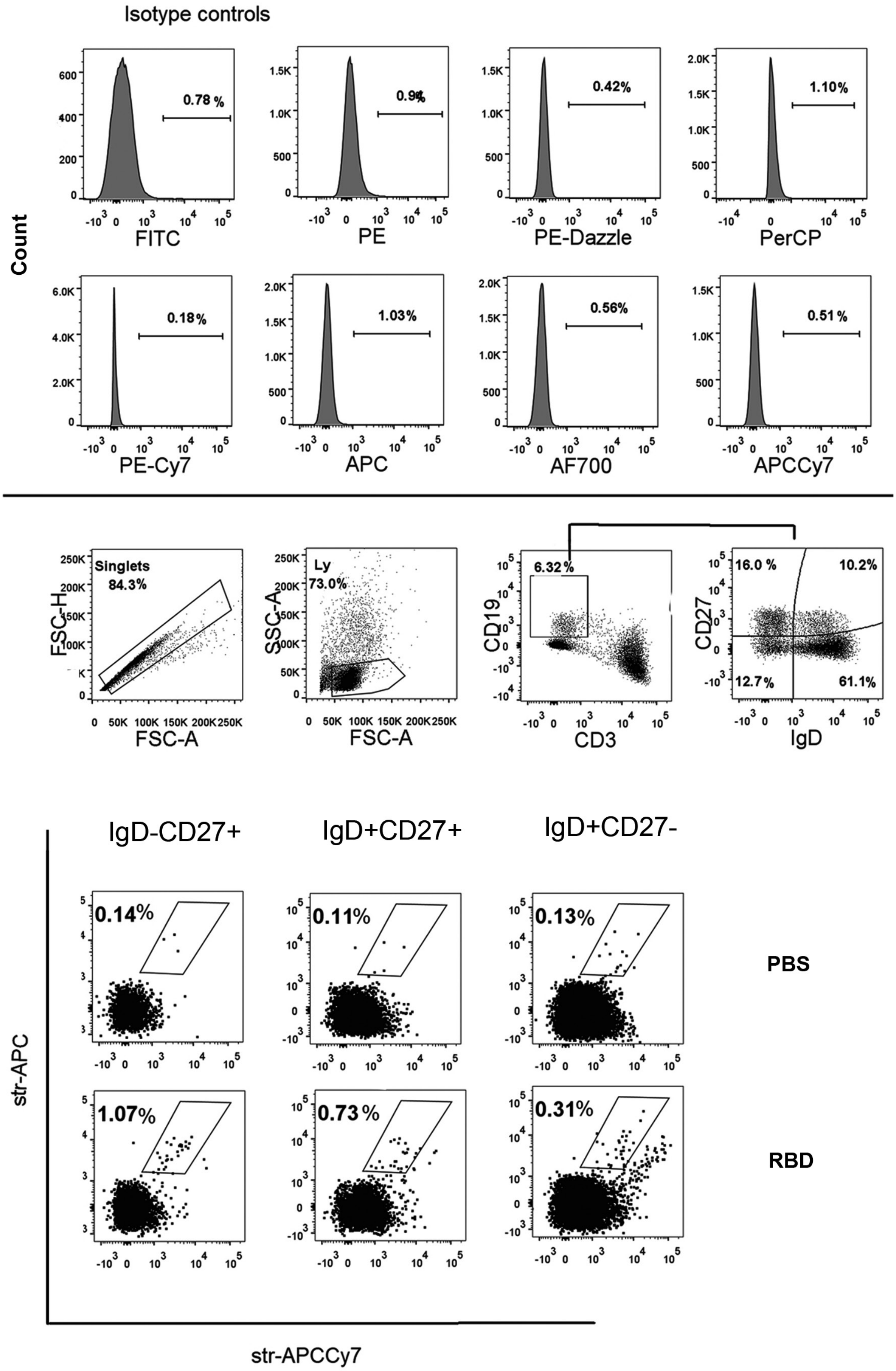

Fig. 1: receptor-binding domain (RBD)-specific B cells - gating strategy and representative flow cytometric plots. Identification and phenotyping of RBD-specific B cells within peripheral blood mononuclear cells (PBMC) isolated from two patients groups [severe acute respiratory syndrome coronavirus 2 (SARS-CoV-2) and SARS-CoV-2+TS] was performed upon treatment of PBMC with biotin-labelled RBD tetramers, followed by incubation with Streptavidin conjugated to two different fluorochromes [allophycocyanin (APC) and APC cyanine7 (APC Cy7)]. Cells were gated to total $CD3^-CD19^+$ to define total B cells. To identify B cell subpopulations, gating was based on IgD and CD27 expression, leading to the classification of four subsets: naïve B cells (IgD^+CD27^-), pre-switch-memory (IgD^+CD27^+), post-switch memory (IgD^-CD27^+) and double-negative/exhausted (DN, IgD^-CD27^-) B cells. RBD-specific B cells were then gated within the first three populations.

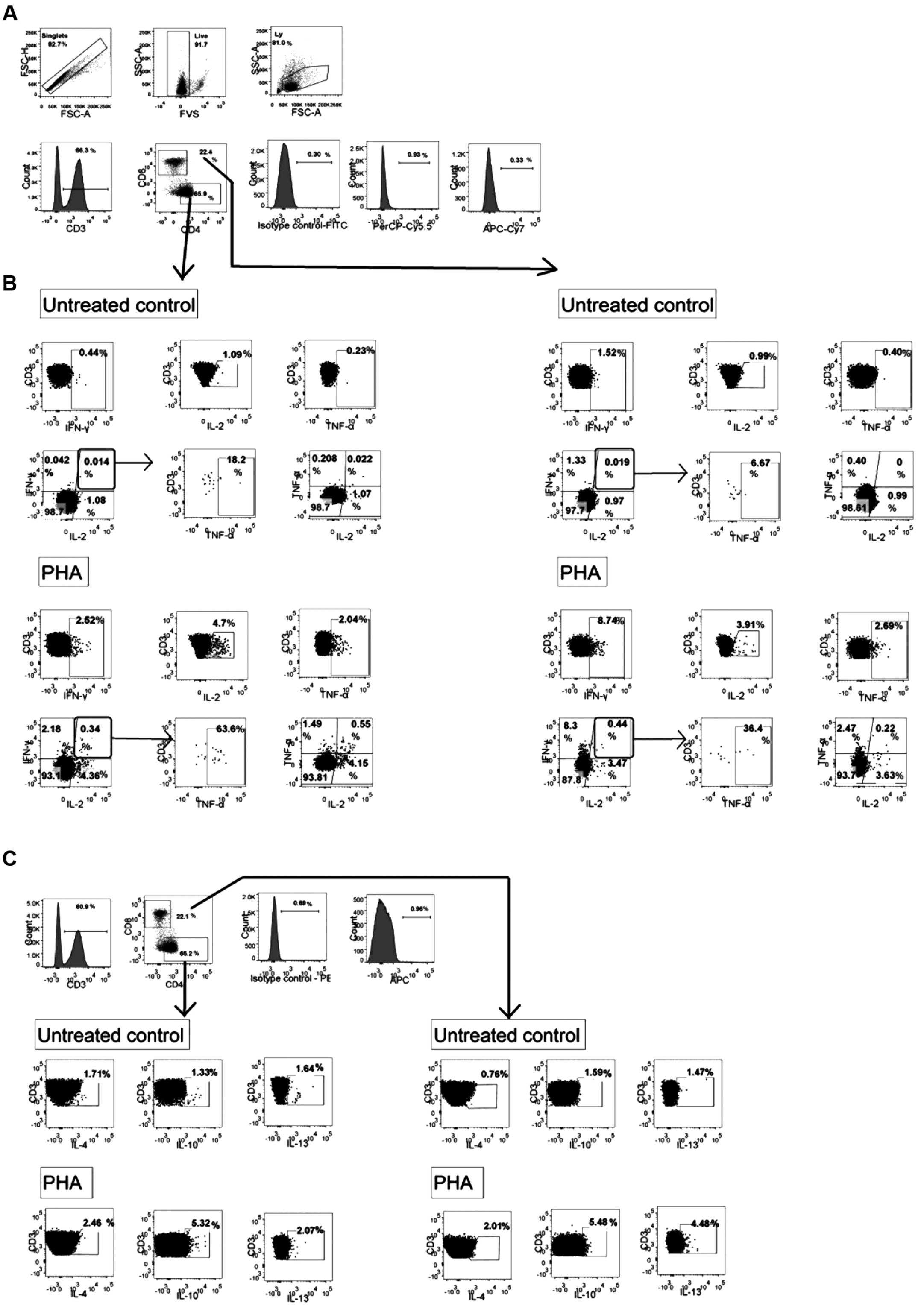

Fig. 2: representative gating strategy of T cells. (A) Exclusion of doublets [FSCA/SSCA - doublets were excluded based on the forward scatter (FSC-H, FSC-A) and cell morphology by FSC-A and side scatter (SSC-A)], dead cells [according to fixable viability stain (FVS)620 staining in independent samples] and cell morphology (FSCA/SSCA) are shown. The cells were gated to total $CD3^+$ cells and within them $CD4^+$ and $CD8^+$. Isotype controls antibodies conjugated with fluorescein isothiocyanate (FITC), peridinin chlorophyll protein/Cyanine (PerCP-Cy) 5.5 and allophycocyanin/Cyanine7 (APC-Cy7) channels were used for determining the expression of interferon (IFN)-γ, interleukin (IL)-2 and tumour necrosis factor (TNF)-α respectively. (B) $CD4^+$ and $CD8^+$ T cells were further analysed for the single, dual or triple expression of cytokines IFN-γ, IL-2 and TNF-α as indicated, either in untreated or the cells treated with phytohaemagglutinin PHA (2 µg/mL) for 6h in the presence of Brefeldin A (1 µM). (C) The same gating strategy was used for the analysis of IL-13, IL-4 and IL-10 within $CD4^+$ and $CD8^+$ T cells, and the percentage of positive cells was determined according to isotype controls in phycoerythrin (PE), PerCP-Cy5.5 and allophycocyanin (APC), respectively.

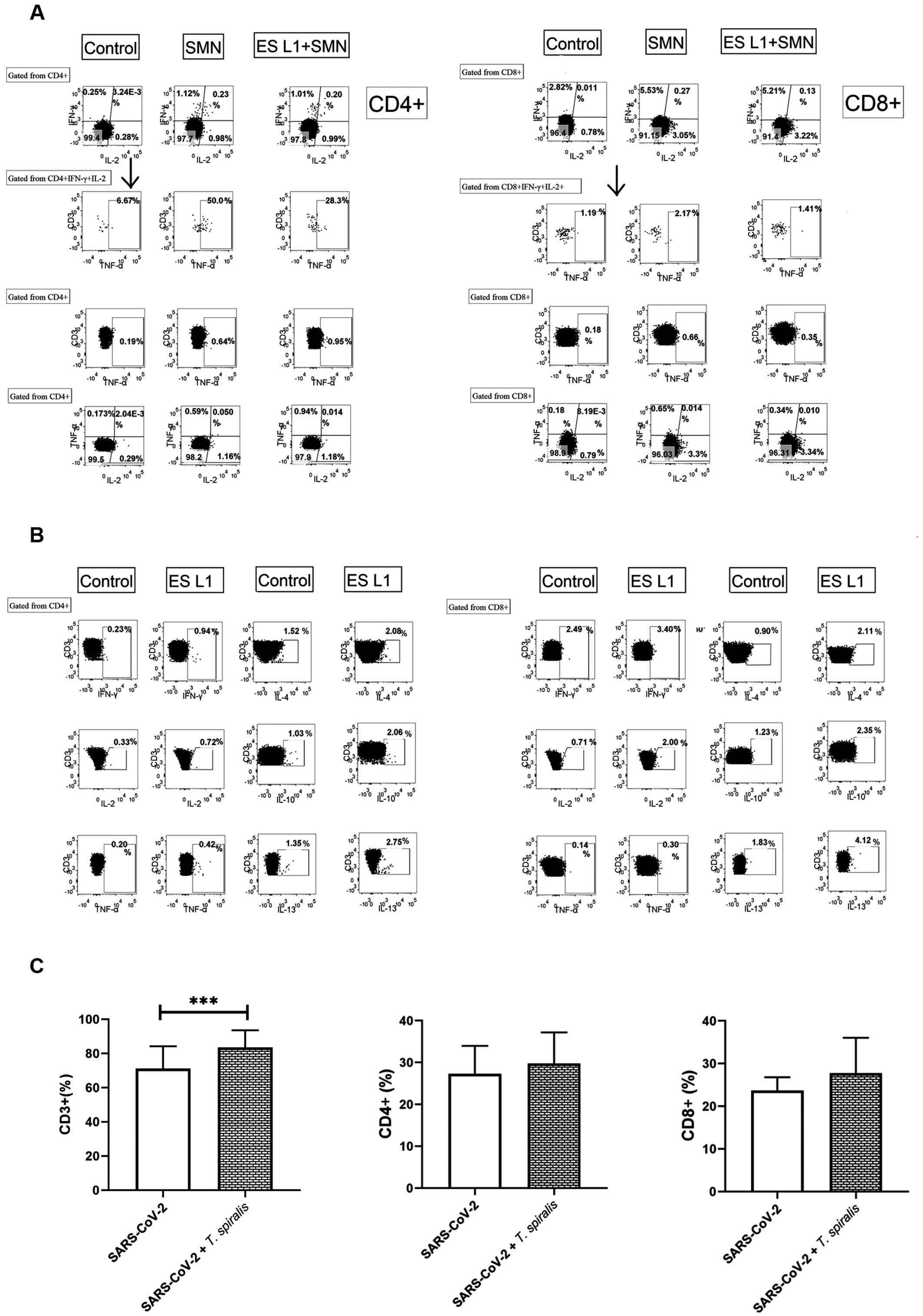

Fig. 3: cytokine expressing T cells - representative flow cytometric plots for phenotyping. Identification and phenotyping of cytokine producing $CD4^+$ and $CD8^+$ T cells within peripheral blood mononuclear cells (PBMCs) isolated from two groups of patients [severe acute respiratory syndrome coronavirus 2 (SARS-CoV-2) and SARS-CoV-2+TS]. (A) Cells were stimulated with SARS-CoV-2 15-mer peptides (SMN) for 6 h at 1 μg/mL in the presence of Brefeldin A (1 uM), with or without prior overnight stimulation with excretory-secretory (ES) L1 (10 ug/mL). Representative plots display the expression of interferon (IFN)-γ, interleukin (IL)-2 and tumour necrosis factor (TNF)-α. To identify dual cytokine producers, CD^{4+} or $CD8^+$ gated cells were further gated on IFN-γ and IL-2 expression. Subsequently, IFN-γ^+IL-2^+ cells were than gated on TNF-α to identify IFN-γ^+IL-2^+TNF-α^+ populations; (B) Cells were incubated overnight with ES L1 (10 μg/mL), while control cells were maintained in medium alone. Representative plots display the expression of IFN-γ, IL-2, TNF-α, IL-4, IL-10 and IL-13; (C) Percentages of $CD3^+$, $CD4^+$ and $CD8^+$ T cells from PBMCs of the two patient groups are shown.
